# Upregulated long noncoding RNA LOC105375913 induces tubulointerstitial fibrosis in focal segmental glomerulosclerosis

**DOI:** 10.1038/s41598-018-36902-2

**Published:** 2019-01-24

**Authors:** Runhong Han, Shuai Hu, Weisong Qin, Jingsong Shi, Caihong Zeng, Hao Bao, Zhihong Liu

**Affiliations:** 1 National Clinical Research Center of Kidney Diseases, Jinling Hospital, Nanjing University School of Medicine, Nanjing, 210002 China; 2 School of Medicine, Southeast University, Nanjing, 210009 China

## Abstract

Tubulointerstitial fibrosis impacts renal prognosis of focal segmental glomerulosclerosis (FSGS). Based on transcriptomic analysis, we found that the level of LOC105375913 was increased in tubular cells of FSGS patients. C3a induced the expression of LOC105375913, which promoted the expression of fibronectin and collagen I in tubular cells. Silence of snail reversed the level of fibronectin and collagen I in cells overexpressing LOC105375913. MiR-27b was predicted and confirmed to regulate the expression of snail in tubular cells, and LOC105375913 contained the response element of miR-27b. The competitive binding between LOC105375913 and miR-27b increased the level of snail and promoted fibrogenesis in tubular cells. Upstream, p38 and XBP-1s regulated the expression of LOC105375913. Inhibition of p38 or silence of XBP-1s decreased the level of LOC105375913, and suppressed the expression of snail, fibronectin and collagen I in tubular cells treated with C3a. Overexpression of LOC105375913 decreased the level of miR-27b, increased the level of snail and caused tubulointerstitial fibrosis in mice. In conclusion, the activation of C3a/p38/XBP-1s pathway induces the expression of LOC105375913 in tubular cells, and LOC105375913 increases the level of snail and induces tubulointerstitial fibrosis through competitive binding of miR-27b in tubular cells of FSGS patients.

## Introduction

Focal segmental glomerulosclerosis (FSGS) accounts 40% of cases of nephrotic syndrome in adults^1^. Tubulointerstitial fibrosis is an independent risk factor of renal function decline in FSGS patients^2,3^. We completed a transcriptome analysis of tubulointerstitial tissues in 5 patients with FSGS and 5 normal controls. Among the differentially expressed long noncoding RNAs (lncRNAs), the level of LOC105375913 showed the maximum increase in tubulointerstitial tissues of FSGS patients.

LncRNAs are a kind of noncoding RNAs longer than approximately 200 nucleotides with no protein-encoding capacity^4^. Emerging evidence suggests that lncRNAs are involved in the regulation of renal fibrosis. Sun J *et al*. reported that 24 lncRNAs were up-regulated in the renal tissues of rats with unilateral ureteral obstruction, and 19 of them were predicted containing Smad3 binding motifs in the promoter^5^. Wang M *et al*. reported that lncRNA CYP4B1-PS1-001 was downregulated in early diabetic nephropathy, and overexpression of CYP4B1-PS1-001 inhibited proliferation and fibrosis of mesangial cells under high glucose conditions^6^. So far, the role of lncRNAs in renal fibrosis of FSGS patients have not been reported.

During nephrotic syndrome, plasma protein leakage into the urinary space directly contributes to local tissue injury. Deposits of the complement component 3 protein (C3) were detected on the proximal tubules of kidneys from nephrotic patients^3^. We noted that the level of C3 mRNA was also upregulated in the tubulointerstitial tissues of FSGS patients. Therefore, both systemic and local complement synthesis contribute to intrarenal complement activation. Tang Z *et al*. reported that the kidneys of C3aR-deficient mice had less interstitial collagen I and alpha-smooth muscle actin in the adriamycin-induced proteinuria model^7^. Zhou M *et al*. reported that C3a induced epithelial-to-mesenchymal transition in proximal tubular epithelial cells on microfluidic devices^8^.

Based on our findings, we believe that the increase in LOC105375913 may be associated with the abnormal exposure of renal tubular cells to complement components in FSGS patients. In this study, experiments were further conducted to investigate the underlying mechanism of LOC105375913 expression and investigate the role of LOC105375913 in the tubular cells of FSGS patients.

## Results

### LOC105375913 is increased in tubular cells and relates to tubulointerstitial fibrosis in FSGS patients

**Figure 1:**
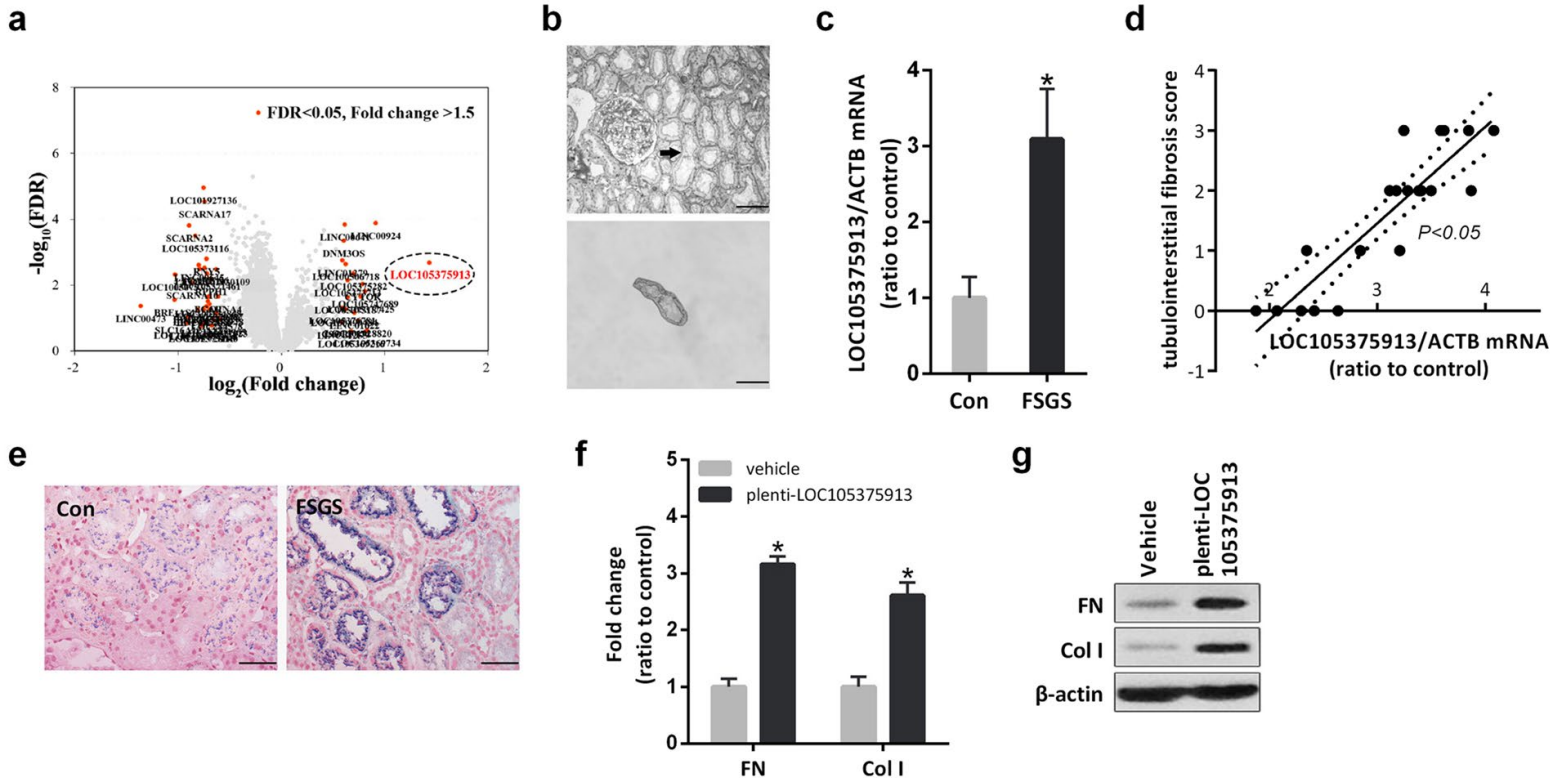
Change of LOC105375913 in renal tubulointerstitial tissues of FSGS patients. (**a**) Volcano plot of differentially expressed lncRNAs in tubulointerstitial tissues of FSGS patients with cutoff values of fold change > 1.5 and FDR < 0.05 (n = 5); (**b**) Isolation of tubulointerstitial tissues by laser capture microdissection technique; (**c**) RT-PCR analysis of LOC105375913 level in another set of 20 FSGS patients and 20 controls; (**d**) Correlation between the level of LOC105375913 and the tubulointerstitial fibrosis score in another set of 20 FSGS patients; (**e**) *In situ* hybridization analysis of LOC105375913 in FSGS patients and normal controls (n = 5); (**f**) RT-PCR analysis of FN and Col I in HK-2 cells transfected with plenti-CMV-LOC105375913 plasmid (n = 5); (**g**) Western blot analysis of FN and Col I in HK-2 cells transfected with plenti-CMV-LOC105375913 plasmid (n = 3). Bar = 20 μm. For statistical analysis, a two-tailed Student’s t-test was used for (**c** and **f**) and spearman correlation was used for (**d**). *P < 0.05 compared with control.

Renal tubulointerstitial tissues from 5 patients with FSGS and 5 normal controls were micro-dissected, and an Affymetrix HTA 2.0 microarray was used to perform a global analysis of the gene expression patterns in the tissues. Among the differentially expressed lncRNAs, the level of LOC105375913 showed the maximum increase in patients with FSGS (Fig. 1a). PCR analysis of another set of patients confirmed that the level of LOC105375913 was significantly increased in the tubulointerstitial tissues of FSGS patients, and was correlated with the score of tubulointerstitial fibrosis in patients with FSGS (Fig. 1b–d). ISH staining showed that LOC105375913 expression was upregulated in the tubular cells of FSGS patients (Fig. 1e). HK-2 cells were then cultured and treated *in vitro*. Overexpression of LOC105375913 obviously increased the mRNA and protein levels of fibronectin (FN) and collagen I (Col I) in HK-2 cells (Fig. 1f,g).

### C3a induces the expression of LOC105375913 in tubular cells

**Figure 2:**
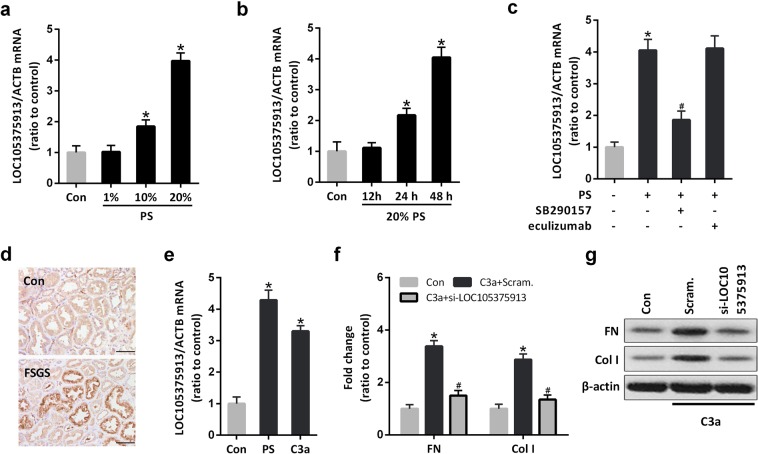
Effect of C3a on the expression of LOC105375913 in tubular cells. (**a**) RT-PCR analysis of LOC105375913 in HK-2 cells treated with different concentrations of patients’ serum (PS) for 48 h (n = 5); (**b**) RT-PCR analysis of LOC105375913 in HK-2 cells treated with 20% PS for different times (n = 5); (**c**) RT-PCR analysis of LOC105375913 in HK-2 cells treated with 20% PS, eculizumab and SB290157 for 48 h (n = 5); (**d**) IHC analysis of C3a in FSGS patients and normal controls (n = 5); (**e**) RT-PCR analysis of LOC105375913 in HK-2 cells treated with 20% PS and C3a for 48 h (n = 5); (**f**) RT-PCR analysis of FN and Col I in HK-2 cells treated with C3a and si-LOC105375913 (n = 5); (**g**) Western blot analysis of FN and Col I in HK-2 cells treated with C3a and si-LOC105375913 (n = 3). Bar = 20 μm. For statistical analysis, one-way ANOVA with Tukey’s post hoc test was used for (**a**–**c**, **e** and **f**). *P < 0.05 compared with control. ^#^*P* < 0.05 compared with PS or C3a-treated cells.

Treatment with patients’ serum significantly induced the expression of LOC105375913 in a time- and dose-dependent manner (Fig. 2a,b). The level of LOC105375913 was significantly increased in HK-2 cells treated with 10% or 20% serum for more than 24 h.

Inhibition of C3aR but not C5b-9 inhibition prevented the increase of LOC105375913 in tubular cells treated with patients’ serum (Fig. 2c). Immunohistochemistry staining (IHC) showed that massive C3a bound to the tubular cells in FSGS patients (Fig. 2d). Treatment with C3a alone induced the overexpression of LOC105375913 in tubular cells (Fig. 2e). C3a treatment also increased the levels of FN and Col I in tubular cells, which was suppressed in cells transfected with LOC105375913 siRNA (Fig. 2f,g).

### LOC105375913 overexpression increases the expression of snail in tubular cells

**Figure 3:**
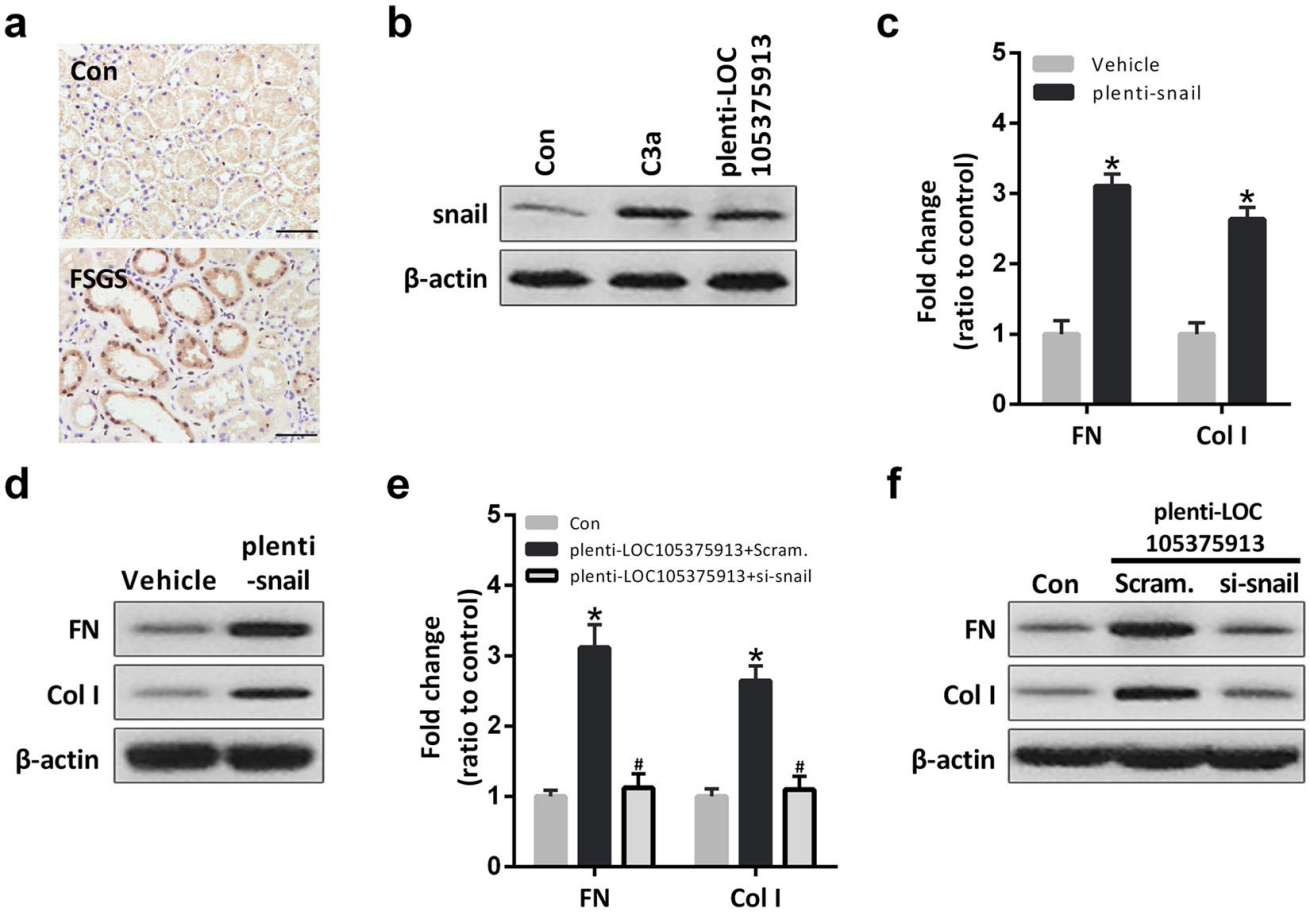
Effect of LOC105375913 on the expression of snail in tubular cells. (**a**) IHC staining of snail in renal tissue of FSGS patients and normal controls (n = 5); (**b**) Western blot analysis of snail in HK-2 cells treated with C3a and plenti-CMV-LOC105375913 plasmid (n = 3); (**c**) RT-PCR analysis of FN and Col I in HK-2 cells transfected with plenti-CMV-snail plasmid (n = 5); (**d**) Western blot analysis of FN and Col I in HK-2 cells transfected with plenti-CMV-snail plasmid (n = 3); (**e**) RT-PCR analysis of FN and Col I in HK-2 cells transfected with LOC105375913 plasmid and si-snail (n = 5); (**f**) Western blot analysis of FN and Col I in HK-2 cells transfected with LOC105375913 plasmid and si-snail (n = 3). For statistical analysis, a two-tailed Student’s t-test was used for (c), and one-way ANOVA with Tukey’s post hoc test was used for (**e**). *P < 0.05 compared with control. ^#^*P* < 0.05 compared with cells transfected with LOC105375913 plasmid.

Snail is a key transcription factor which involved in tubulointerstitial fibrosis in renal disease. IHC analysis showed that, the expression of snail was increased in the tubular cells of FSGS patients, compared to normal controls (Fig. 3a). Treatment with C3a or transfection of LOC105375913 plasmid increased the expression of snail in tubular cells (Fig. 3b).

Overexpression of snail increased the level of FN and Col I in tubular cells, similar to the effect of C3a treatment or LOC105375913 overexpression (Fig. 3c,d). Conversely, silence of snail reversed the level of FN and Col I in tubular cells transfected with LOC105375913 plasmid (Fig. 3e,f).

### LOC105375913 upregulates the level of snail by competitive binding of miR-27b in tubular cells

**Figure 4:**
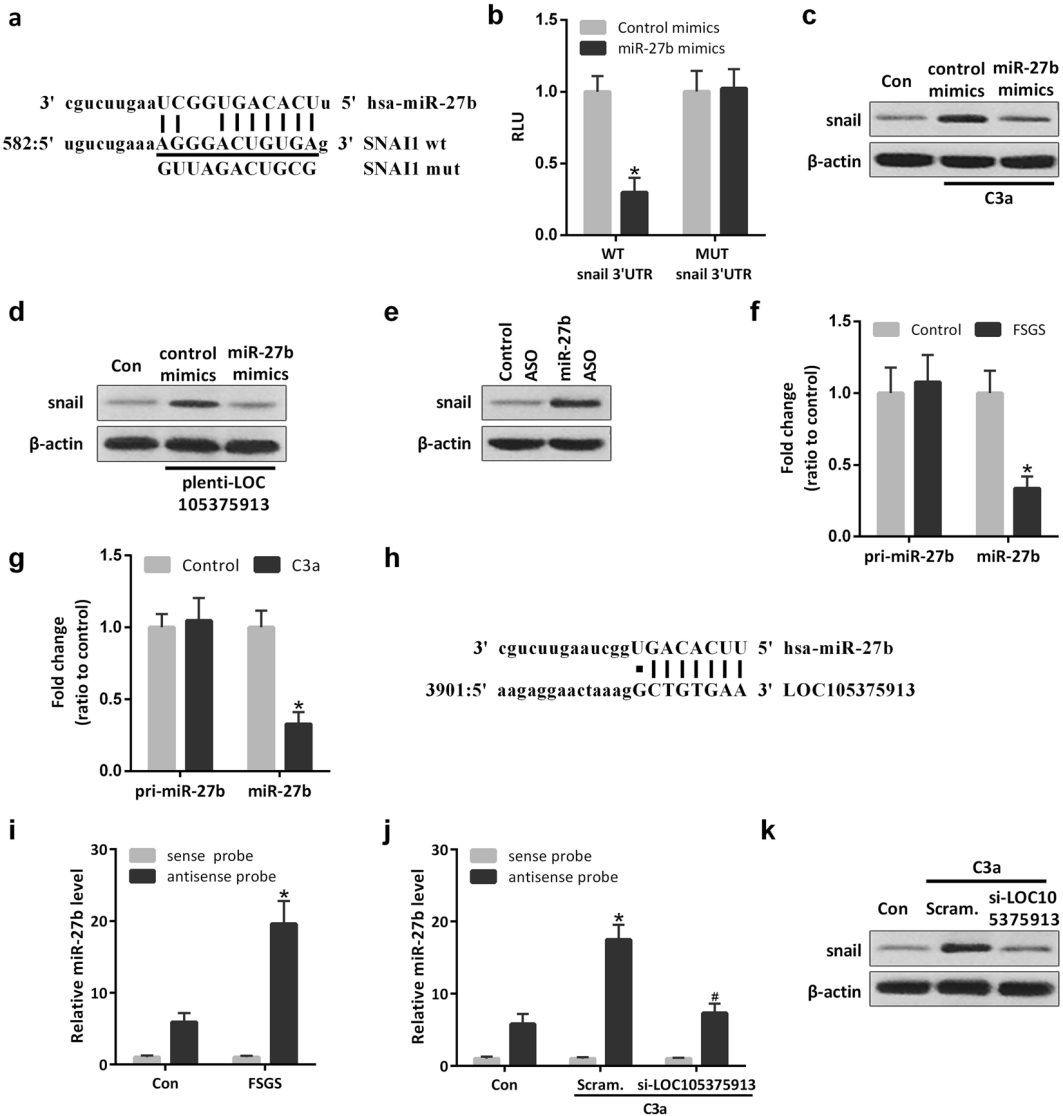
LOC105375913 upregulates snail by competitive binding of miR-27b in tubular cells. (**a**) The complementarity map between miR-27b and snail 3′ UTR sequence; (**b**) Normalized luciferase activity of reporter constructs containing the 3′ UTR of snail or mutant 3′ UTR of snail in HK-2 cells cotransfected with miR-27b mimics (n = 5); (**c**) Western blot analysis of snail in HK-2 cells treated with C3a and miR-27b mimics (n = 3); (**d**) Western blot analysis of snail in HK-2 cells transfected with LOC105375913 plasmid and miR-27b mimics (n = 3); (**e**) Western blot analysis of snail in HK-2 cells transfected with miR-27b ASO (n = 3); (**f**) RT-PCR analysis of pri-miR-27b and miR-27b in FSGS patients and normal controls (n = 20); (**g**) RT-PCR analysis of pri-miR-27b and miR-27b in HK-2 cells treated with C3a (n = 5); (**h**) The binding site in the LOC105375913 targeted by miR-27b; (**i**) RNA pull-down and RT-PCR analysis of the binding between LOC105375913 and miR-27b in tubulointerstitial tissues of FSGS patients (n = 5); (**j**) RNA pull-down and RT-PCR analysis of the binding between LOC105375913 and miR-27b in HK-2 cells treated with C3a and si-LOC105375913 (n = 5); (**k**) Western blot analysis of snail in HK-2 cells treated with C3a and si-LOC105375913 (n = 3). For statistical analysis, one-way ANOVA with Tukey’s post hoc test was used for (**b**,**i** and **j**) and a two-tailed Student’s t-test was used for (**f** and **g**). *P < 0.05 compared with control; ^#^*P* < 0.05 compared with C3a-treated cells.

Among the most enriched miRNAs in renal tissues, computational analysis detected a miR-27b binding sequence in the 3′UTR of snail mRNA (Fig. 4a)^9^. MiR-27b mimics significantly inhibited the reporter activity of Luc-snail 3′UTR. Site-directed mutations rescued the miR-27b-mediated inhibition of Luc-snail 3′UTR (Fig. 4b). Transfection with miR-27b mimics also reversed the expression of snail in tubular cells treated with C3a or transfected with LOC105375913 plasmid (Fig. 4c,d). Conversely, transfection with miR-27b antisense oligonucleotide (ASO) increased the level of snail in HK-2 cells (Fig. 4e). PCR analysis showed that the level of miR-27b but not pri-miR-27b was decreased in the tubulointerstitial tissues of FSGS patients and in tubular cells treated with C3a, suggesting a post-transcriptional mechanism involved in the regulation of miR-27b in tubular cells of FSGS (Fig. 4f,g).

Bioinformatic analysis with RNAHybrid software showed that, LOC105375913 contains the response element of miR-27b (Fig. 4h). RNA pull-down and PCR analysis showed that miR-27b directly bound to LOC105375913 in tubulointerstitial tissues, and this binding was significantly increased in the tissues of FSGS patients compared to normal controls (Fig. 4i). Treatment with C3a increased the binding between LOC105375913 and miR-27b, which was prevented by LOC105375913 siRNA (Fig. 4j). Silence of LOC105375913 also decreased the level of snail in C3a-treated tubular cells (Fig. 4k).

### C3a induces the expression of LOC105375913 by activating p38 MAPK pathway in tubular cells

**Figure 5:**
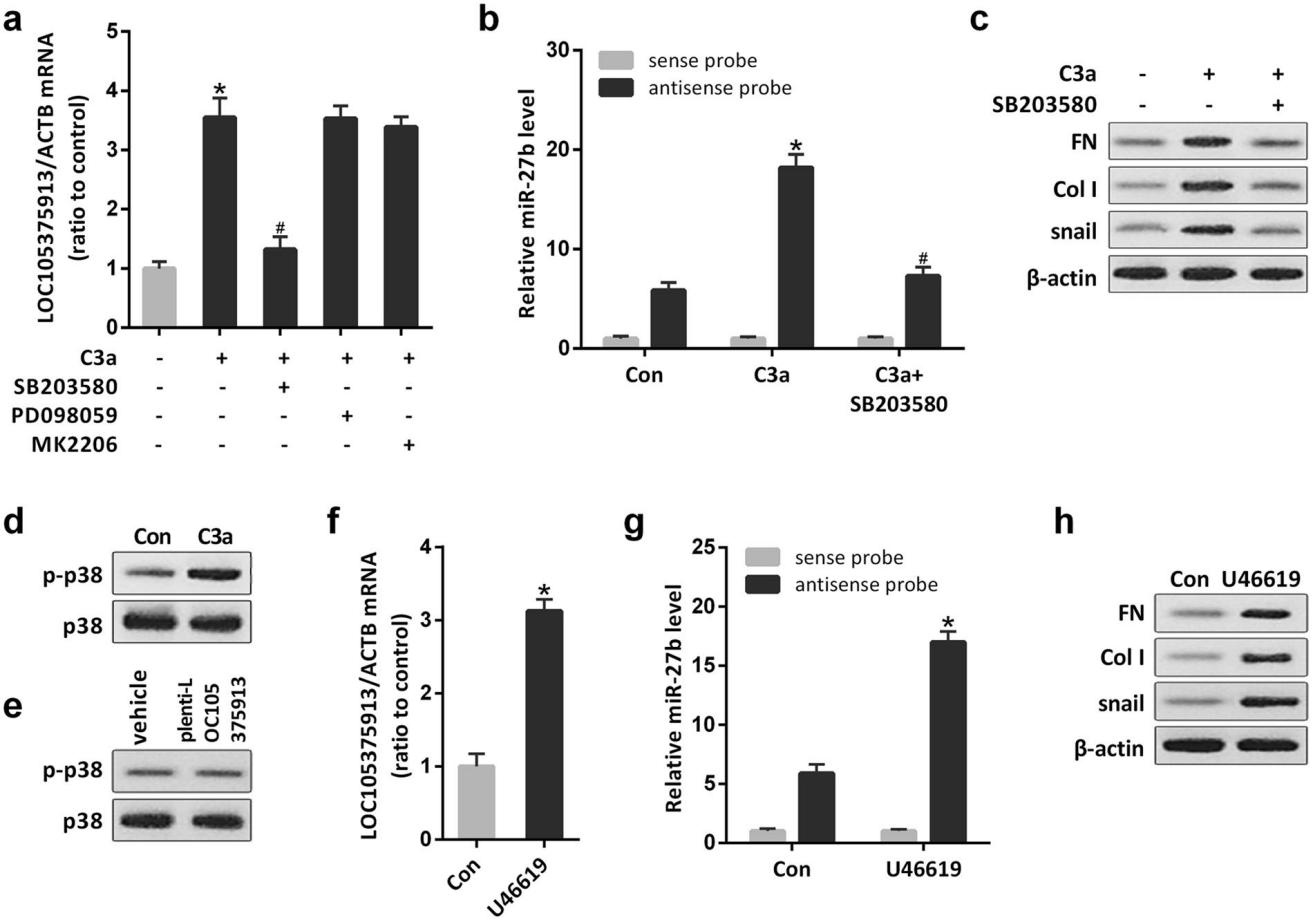
Role of p38 MAPK in the expression of LOC105375913 in tubular cells. (**a**) RT-PCR analysis of LOC105375913 in HK-2 cells treated with C3a, SB203580, PD098059 and MK2206 (n = 5); (**b**) RNA pull-down and RT-PCR analysis of the binding between LOC105375913 and miR-27b in HK-2 cells treated with C3a and SB203580 (n = 5); (**c**) Western blot analysis of snail, FN and Col I in HK-2 cells treated with C3a and SB203580 (n = 3); (**d,e**) Western blot analysis of p-p38 in HK-2 cells treated with C3a or plenti-CMV-LOC105375913 plasmid (n = 3); (**f**) RT-PCR analysis of LOC105375913 in HK-2 cells treated with U46619 (n = 5); (**g**) RNA pull-down and RT-PCR analysis of the binding between LOC105375913 and miR-27b in HK-2 cells treated with U46619 (n = 5); (**h**) Western blot analysis of snail, FN and Col I in HK-2 cells treated with U46619 (n = 3). For statistical analysis, one-way ANOVA with Tukey’s post hoc test was used for (**a**,**b** and **g**) and a two-tailed Student’s t-test was used for (**f**). *P < 0.05 compared with control. ^#^*P* < 0.05 compared with C3a-treated cells.

Protein kinase B (AKT), p38 and extracellular signal-regulated kinase (ERK) function downstream of C3aR. The p38 inhibitor SB203580 but not the AKT inhibitor MK2206 or the ERK inhibitor PD098059 significantly prevented the upexpression of LOC105375913 in tubular cells treated with C3a (Fig. 5a). The p38 inhibitor SB203580 also suppressed the binding between LOC105375913 and miR-27b, and decreased the level of snail, FN and Col I in tubular cells treated with C3a (Fig. 5b,c).

Treatment with C3a induced the phosphorylation of p38 in tubular cells, while overexpression of LOC105375913 didn’t change the level of p-p38 in tubular cells (Fig. 5d,e). Conversely, treatment with p38 activator induced the expression of LOC105375913, promoted the binding between LOC105375913 and miR-27b, and increased the level of snail, FN and Col I in tubular cells (Fig. 5f–h).

### Transcription factor XBP-1s regulates the expression of LOC105375913 in tubular cells

**Figure 6:**
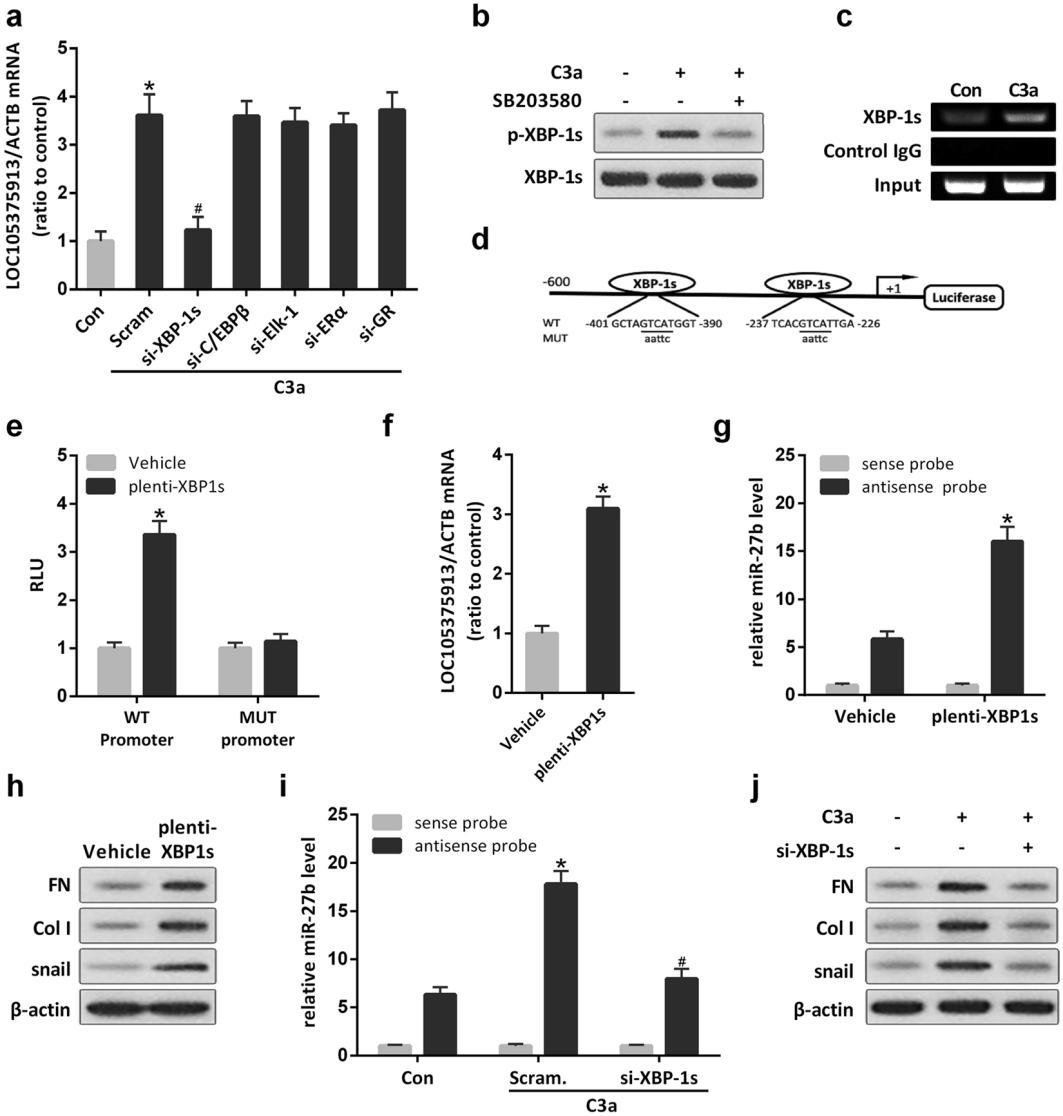
Role of XBP-1s in the expression of LOC105375913 in tubular cells. (**a**) RT-PCR analysis of LOC105375913 in HK-2 cells treated with C3a, XBP-1s siRNA, C/EBPβ siRNA, Elk-1 siRNA, ERα siRNA and GR siRNA (n = 3); (**b**) Level of XBP-1s and p-XBP-1s protein in HK-2 cells treated with C3a and SB203580 (n = 3); (**c**) ChIP analysis of the binding between XBP-1s and LOC105375913 promoter in HK-2 cells treated with C3a (n = 3); (**d**) Schematic of the constructed LOC105375913 promoter-luciferase reporter plasmids; (**e**) Normalized luciferase activity of reporter constructs in HK-2 cells cotransfected with XBP-1s plasmid (n = 5); (**f**) Level of LOC105375913 in HK-2 cells transfected with XBP-1s plasmid (n = 5); (**g**) RNA pull-down and RT-PCR analysis of the binding between LOC105375913 and miR-27b in HK-2 cells transfected with XBP-1s plasmid (n = 5); (**h**) Western blot analysis of snail, FN and Col I in HK-2 cells transfected with XBP-1s plasmid (n = 3); (**i**) RNA pull-down and RT-PCR analysis of the binding between LOC105375913 and miR-27b in HK-2 cells treated with C3a and XBP-1s siRNA (n = 5); (**j**) Western blot analysis of snail, FN and Col I in HK-2 cells treated with C3a and XBP-1s siRNA (n = 3). For statistical analysis, one-way ANOVA with Tukey’s post hoc test was used for (**a**,**e**,**g** and **i**), and a two-tailed Student’s t-test was used for (**f**). *P < 0.05 compared with control; ^#^*P* < 0.05 compared with C3a-treated cells.

The PROMO (TRANSFAC 8.3) software was applied to explore the transcription factors that may bind to the LOC105375913 promoter region. Among the predicted transcriptional factors, 5 factors have been reported to be the downstream substrates of p38 MAPK in the literature^10^. We knocked down the expression level by siRNAs, and found that knockdown of XBP-1s significantly inhibited the increase of LOC105375913 in HK-2 cells treated with C3a (Fig. 6a). The phosphorylation of XBP-1s was increased in tubular cells treated with C3a, and inhibition of p38 prevented the C3a-induced phosphorylation of XBP-1s (Fig. 6b).

ChIP analysis confirmed that XBP-1s bound to the LOC105375913 promoter in tubular cells, which was enhanced by C3a treatment (Fig. 6c). Overexpression of XBP-1s increased the expression of luciferase reporter construct containing the binding sequence. Site-directed mutations rescued the XBP-1s-mediated upregulation of the LOC105375913 promoter-luciferase reporter plasmid (Fig. 6e). XBP-1s overexpression also increased the level of endogenous LOC105375913, promoted the binding between LOC105375913 and miR-27b, and increased the expression of snail, FN and Col I in tubular cells (Fig. 6f–h). Conversely, knockdown of XBP-1s suppressed C3a-induced the binding between LOC105375913 and miR-27b, and decreased the expression of snail, FN and Col I in tubular cells (Fig. 6i,j).

### Overexpression of LOC105375913 induces tubulointerstitial fibrosis in mice

**Figure 7:**
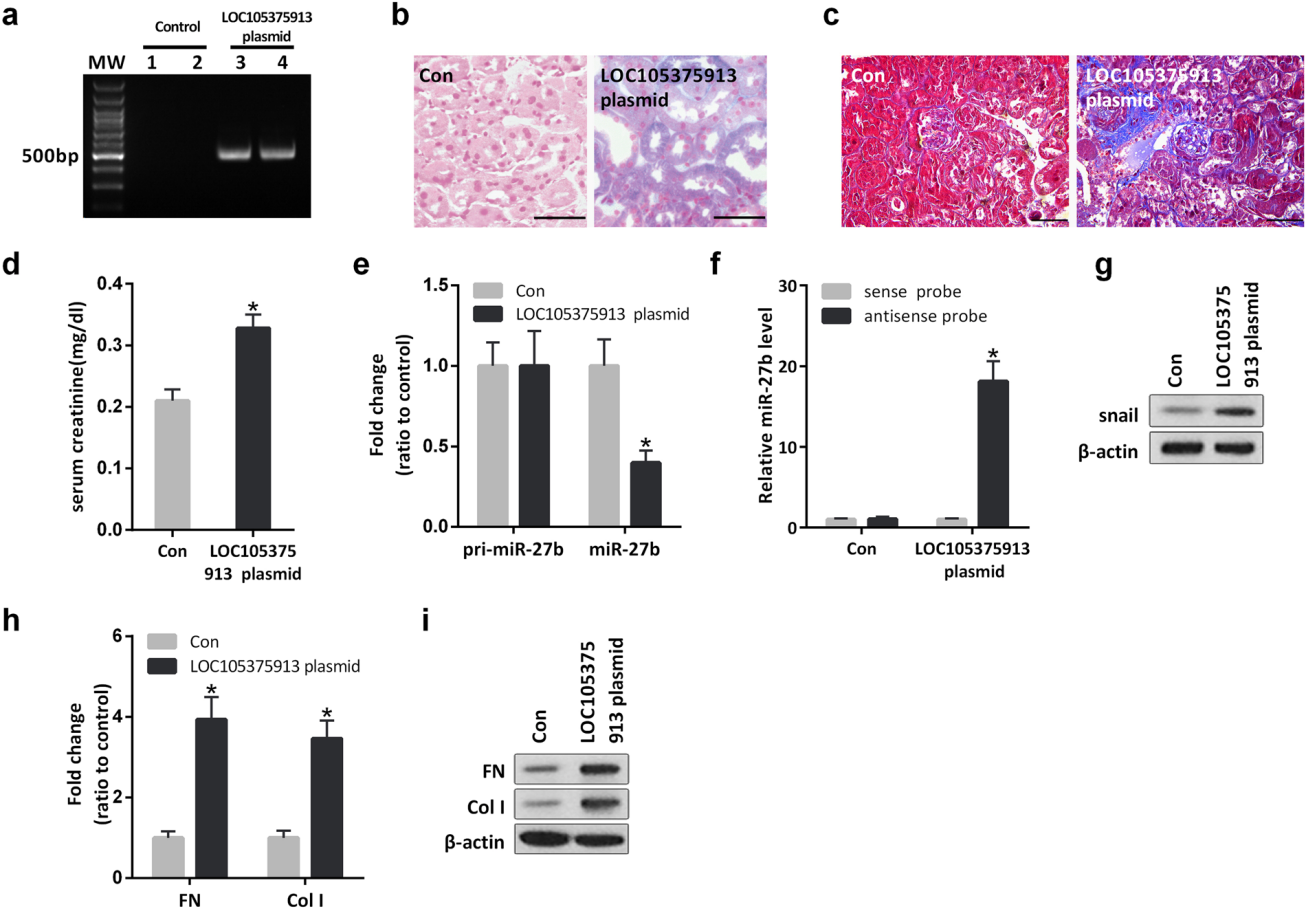
Effect of LOC105375913 overexpression on tubulointerstitial fibrosis in mice. (**a**) PCR analysis of LOC105375913 in tubulointerstitial tissues of mice treated with control plasmid or LOC105375913-expressing plasmid; (**b**) ISH analysis of LOC105375913 in tubulointerstitial tissues of mice (n = 6); (**c**) Masson staining of renal sections in mice (n = 6); (**d**) Level of serum creatinine in mice (n = 6); (**e**) Level of pri-miR-27b and miR-27b in tubulointerstitial tissues of mice (n = 6); (**f**) RNA pull-down and RT-PCR analysis of the binding between LOC105375913 and miR-27b in tubulointerstitial tissues of mice (n = 6); (**g**) Western blot analysis of snail in tubulointerstitial tissues of mice (n = 6); (**h**) RT-PCR analysis of FN and Col I in tubulointerstitial tissues of mice (n = 6); (**i**) Western blot analysis of FN and Col I in tubulointerstitial tissues of mice (n = 6). For statistical analysis, a two-tailed Student’s t-test was used for (**d**,**e** and **h**), and one-way ANOVA with Tukey’s post hoc test was used for (**f**). *P < 0.05 compared with mice treated with control plasmid. Bar = 20 μm.

LOC105375913 was not conserved in mouse. To analyze the pathogenic role of LOC105375913 *in vivo*, we elevated renal LOC105375913 expression by hydrodynamic-based delivery of LOC105375913-expressing plasmid in mice. After 8 weeks of treatment, LOC105375913 was obviously expressed in the tubular cells of mice (Fig. 7a,b). Mice overexpressing LOC105375913 caused obvious tubulointerstitial fibrosis and an increase in serum creatinine (Fig. 7c,d).

Overexpression of LOC105375913 didn’t change the level of pri-miR-27b, instead it decreased the level of miR-27b in the tubulointerstitial tissues of mice (Fig. 7e). RNA pull-down and RT-PCR analysis showed that the binding between LOC105375913 and miR-27b was increased in the tubulointerstitial tissues of mice overexpressing LOC105375913 (Fig. 7f). Meanwhile, overexpression of LOC105375913 increased the expression of snail in tubulointerstitial tissues of mice (Fig. 7g). As a result, the level of FN and Col I were obviously increased in the mice overexpressing LOC105375913 (Fig. 7h,i).

## Discussion

FSGS accounts for about 40% of nephrotic syndrome in adult^1^. Most of the patients suffer non selective proteinuria, hypertension, renal impairment with tubular dysfunction at onset. In pathology, patients with FSGS often show severe renal tubulointerstitial lesions^11^. The prognosis of FSGS patients is poor, with renal survival rate only 43.8% at 10 years after the biopsy. By multivariate proportional analysis, chronic tubulointerstitial injury was one of the independent predictors of end-stage renal disease in FSGS patients^2^.

LncRNA refers to noncoding RNA which has more than 200 nucleotides^12^. LncRNAs plays an important role in the development of renal disease. Puthanveetil P *et al*. reported that lncRNA MALAT1 regulates glucose-induced up-regulation of inflammatory mediators IL-6 and TNF-α through activation of SAA3 in the endothelial cells^13^. Sun SF *et al*. showed that lncRNA Erbb4-IR promotes renal fibrosis by suppressing miR-29b in db/db mice^14^. We completed a tubulointerstitial transcriptional analysis of 5 cases of FSGS and 5 normal controls, the level of LOC105375913 showed the maximum increase in the tubulointerstitial tissues of FSGS patients, and overexpression of LOC105375913 induced the expression of FN and Col I in tubular cells.

Complement activation plays an important role in the progression of chronic kidney disease. In patients with FSGS, the permeability of glomerular filtration membrane increased, and plasma complement protein leaked into the renal tubule lumen. In our transcriptome analysis, the level of C3 mRNA was also increased in tubular tissues of FSGS patients. Therefore, both systemic and local complement synthesis contribute to intrarenal complement protein activation in FSGS patients. Zaferani A *et al*. found that tubular heparan sulfate serves as a docking platform for the alternative complement component properdin in proteinuric renal disease^15^. Properdin stabilizes the alternative pathway convertase that lead to the formation of C3a and C5b-9. Tang Z *et al*. reported that C3a induces tubular epithelial to mesenchymal transition in proteinuric nephropathy^7^. Our results indicate that C3a induces the upexpression of LOC105375913 in tubular cells of FSGS.

Snail is a zinc finger protein, which promotes the epithelial to mesenchymal transition in renal tubular cells^16^. The level of snail expression was upregulated in unilateral ureteral obstruction rat model and TGF-β1-treated renal tubular cells^17^. Knockdown of snail reversed the expression of fibronectin in tubular cells stimulated by TGF-β1. Gu H *et al*. reported that C3a stimulated the expression of snail in small airway epithelial cells^18^. Our staining showed that the expression of snail was increased in renal tubular cells of FSGS patients and the stimulation with C3a led to the increase of snail in HK-2 cells, suggesting that snail is involved in the renal fibrosis of FSGS patients.

Competing endogenous RNA (ceRNA) is an important way for lncRNA to participate in post transcriptional regulation of gene expression^12,19^. Yu F *et al*. reported that lncRNA-p21 inhibits the Wnt/β-Catenin pathway in activated hepatic stellate cells via sponging miR-17-5p^20^. Tao H *et al*. showed that lncRNA GAS5 controls cardiac fibroblast activation and fibrosis by targeting miR-21 via PTEN/MMP-2 signaling pathway^21^. Li X *et al*. reported that lncRNA PFAL promotes lung fibrosis through CTGF by competitively binding miR-18a^22^. Among the 50 miRNAs enriched in renal tissues, miR-27b was predicted and confirmed to target the 3′ UTR region of snail mRNA^9^. Previously, Graham JR *et al*. reported that TGF-β1 promoted fibrosis process by inhibiting miR-27b expression in alveolar epithelial cells^23^. Our analysis showed that miR-27b response element existed in the sequence of LOC105375913. Overexpression of LOC105375913 decreased the level of miR-27b, increased the level of snail and caused tubulointerstitial fibrosis in mice.

Activation of p38 MAPK has been reported to be involved in renal fibrosis by independent studies^24,25^. Our experiments showed that inhibition of p38 MAPK reversed the upregulation of LOC105375913 in C3a-treated tubular cells. We explored and confirmed that, the activation of p38 phosphorylated XBP-1s, which bound to the promoter and increased the expression of LOC105375913 in tubular cells. Inhibition of p38 or silence of XBP-1s decreased the level of LOC105375913, suppressed the binding between LOC105375913 and miR-27b, decreased the level of snail, FN and Col I in tubular cells treated with C3a. In consistent with our findings, Zhao S *et al*. reported that XBP-1s is involved in the lipid synthesis in renal tubular epithelial cells treated with high glucose^26^. Mo XT *et al*. reported that XBP-1s participates in snail expression in alveolar epithelial cells^27^.

In conclusion, C3a/p38/XBP-1s pathway induces the expression of LOC105375913 in renal tubular cells, and LOC105375913 promotes the expression of snail and induce fibrogenesis through competitive binding of miR-27b in tubular cells of FSGS patients.

## Methods

### Patients and control subjects

Five FSGS patients who underwent renal biopsies at Jingling Hospital were recruited for tubulointerstitial transcriptome analysis (Table 1). Other 20 FSGS patients were enrolled for validation study. Control tissues were obtained from the unaffected portion of surgical nephrectomies, and were confirmed to be normal through light microscope analysis. The study was carried out in accordance with the principles of the Declaration of Helsinki and was approved by the ethics committees of Jinling Hospital. Renal specimens were kept in Renal Biobank of National Clinical Research Center of Kidney Diseases at Jinling Hospital. Informed consent has been obtained from each participant.

**Table 1:** Demographic characteristics in the discovery and validation cohorts.

Group	Number	eGFR ml/min per 1.73 m^2^	Age (years)	Gender (% female)	Disease course (months)
Discovery cohort					
Normal	5	115.9 ± 18.6	34.1 ± 7.4	20	—
FSGS	5	68.5 ± 28.5	30.6 ± 11.7	20	5.0 (0.6–15.0)
Validation cohort					
Normal	20	112.3 ± 15.9	37.3 ± 9.3	40	—
FSGS	20	57.7 ± 40.3	32.9 ± 17.3	40	5.7 (0.7–11.0)

### Isolation of tubulointerstitial tissues

For array analysis, glomeruli and the corresponding tubulointerstitium were manually separated under a stereomicroscope using 2 dissection needle holders in RNAlater at 4 °C. For PCR analysis, tubulointerstitium were isolated by laser capture microdissection procedure with the Leica AS LMD System (Leica Microsystems AG, Wetzlar, Germany). Approximately 50 cross-sections were captured from each case^28^.

### Gene expression profile analysis

Transcriptomic analysis of microdissected tubulointerstitial tissues were performed with Affymetrix HTA2.0 microarrays in according to standard procedures as described by Affymetrix. The accession number for the microarray data reported in this paper is GEO: GSE121211.

### RT-PCR analysis

Total RNA was extracted using miRNeasy Mini Kit (No. 217004). Reverse transcription was carried out with miScript II RT Kit (No. 218161). QuantiTect SYBR Green PCR Master Mix (No. 204143, Qiagen, Valencia, CA) was used for gene expression level measurement. The primers for PCR analysis were listed in Supplementary Table S1.

### *In situ* hybridization analysis of LOC105375913

LOC105375913 expression was evaluated by *in situ* hybridization (ISH) in paraffin-embedded renal sections. Paraffin tissue sections were deparaffinized with xylene, rehydrated with ethanol dilution series and treated with 15 μg/ml proteinase K at 37 °C for 15 min. Then slides were fixed in 4% paraformaldehyde and hybridized with 5′ digoxin-labeled LOC105375913 probe at 55 °C overnight. After washing, slides were treated with blocking buffer for 30 min. Slides were then incubated with anti-DIG-AP in blocking buffer for 1 h. LOC105375913 was visualized in a staining reaction with NBT/BCIP solution^29–31^.

### Culture and treatment of HK-2 cells

Immortalized tubular epithelial cells (HK-2) were cultured in DMEM/F12 medium supplemented with 10% FBS. After synchronization, cells were treated with 20% FSGS patients’ serum (PS) or 40 nM C3a (204881, Merck-Calbiochem). For intervention studies, 1 μM of C3aR antagonist SB290157 (sc-222291, Santa Cruz), 100 μg/ml of eculizumab (Soliris, Alexion Pharmaceuticals), 10 μM of p38 MAPK inhibitor SB203580 (sc-3533, Santa Cruz), 50 μM of ERK inhibitor PD98059 (sc-3532, Santa Cruz), 10 μM of Akt inhibitor MK2206 (sc-364537, Santa Cruz) or 1 μM of U-46619 (sc-201242, Santa Cruz) was given 30 min before treatments. To infect HK-2 cells with plenti-CMV-LOC105375913, plenti-CMV-snail or plenti-CMV-XBP-1s plasmid, the lentiviral stock was mixed with polybrene (1 µg/ml) and added to cells. C/EBPβ siRNA (sc-44251), Elk-1 siRNA (sc-35290), ERα siRNA (sc-29305), GR siRNA (sc-35505), snail siRNA(sc-38398) and XBP-1s siRNA (sc-38627) were bought from Santa Cruz (Dallas, Texas, USA). LOC105375913 siRNA was bought from Thermo fisher (4390771, Carlsbad, California, USA). Transfection of siRNA, miRNA mimics or miRNA antisense oligonucleotide (ASO) was conducted with Lipofectamine 2000.

### Immunohistochemical staining

Paraffin-embedded sections were deparaffinized and rehydrated. Endogenous peroxidase was blocked with 0.3% hydrogen peroxide in phosphate buffered saline (PBS) for 30 min. The sections were incubated for 1 h at room temperature with primary antibody diluted in 1% BSA in PBS (Supplementary Table S2). The staining was visualized with Polyvalent HRP/DAB detection kit (ab64264, Abcam, Cambridge, USA). Negative controls were obtained by omission of the primary antibody from the staining procedure.

### Western blot analysis

Western blots were performed as previously described^32^. Tissues or cells were lysed in RIPA buffer supplemented with protease inhibitors. Protein concentrations were determined using a bicinchoninic acid protein assay kit (Sigma, St. Louis, MO). 25 μg of the total proteins was loaded into the wells of 10% SDS-PAGE along with the molecular weight markers. After running gel for 1 hour, the proteins were transferred onto PVDF membranes, and the membranes were blocked with 5% skimmed milk in TBST buffer (150 mM NaCl, 20 mM Tri-HCl, pH 7.4, 0.02% Tween 20). Respective primary and secondary antibodies were used to detect the expression of target proteins (Supplementary Table S2).

### Luciferase assay

Cells were cotransfected with the 0.1 μg reporter constructs, 0.02 μg Renilla construct and 50 nM miR-27b mimics. The firefly and renilla luciferase activity were determined using the Dual-Luciferase Reporter Assay System (E1960, Promega). Values were normalized using renilla luciferase^28^.

### Biotin pull-down assay

Tissue or cells were lysed in 10% glycerol, 20 mM Tris (pH 8), 0.2 mM EDTA, 0.5% NP-40, 0.5 M KCl, 1 mM DTT, supplemented with 1 u/µl RNAse OUT (10777019, Invitrogen)^33^. The biotinylated DNA probe complementary to LOC105375913 (100 pmol) was incubated with Dynabeads M-280 Streptavidin (11205D, Invitrogen, CA, USA) at room temperature for 10 min to generate probe-coated beads according to the manufacturer’s protocol. Then, tissue or cell lysates(2 μg) were incubated with the probe-coated beads, and the RNA complexes bound to these beads were extracted for PCR analysis^34,35^.

### ChIP analysis of XBP-1s DNA binding

ChIP assay was performed with the ChIP-IT Express Magnetic Chromatin Immunoprecipitation kit (53008, Active Motif, Carlsbad, CA, USA)^28^. The immune-precipitations were performed with 2 μg antibodies specific for XBP-1s or an IgG negative control at 4 °C overnight with rotation. After de-crosslinking and proteinase treatment, the antibody-associated DNA fragments were subjected to PCR amplification with primer sets that covered the XBP-1s binding element on LOC105375913 promoter (Supplementary Table S1).

### Analysis of phosphorylated XBP-1s with immunoprecipitation

Cells were harvested and lysed in NP-40 buffer supplemented with protease inhibitor. Whole-cell lysates were used for immunoprecipitation with anti XBP-1s antibody. 4 μg of antibody was added to 1 ml of cell lysate, which was incubated at 4 °C for 12 h. After addition of protein A/G agarose beads, the incubation was continued for 1 h. Immunoprecipitates were washed with lysis buffer and analyzed by western blotting with anti-Phospho-Ser/Thr/Tyr antibody.

### Overexpression of LOC105375913 in mice

Animal studies were approved by the Institutional Animal Care and Use Committee of Jinling Hospital. We obtained the male C57BL/6 mice (8 weeks) from the Model Animal Research Center of Nanjing University (Nanjing, China). LOC105375913 was not conserved in mouse. In order to express exogenous LOC105375913 in the kidney of mice, we administered LOC105375913-expressing plasmid to the mice using a previously described hydrodynamic-based gene-transfer technique^9^. Briefly, we mixed LOC105375913-expressing plasmid (20 μg) into approximately 2.6 ml of TransIT-EE Hydrodynamic Delivery Solution (MIR5310, Mirus, Madison, USA). Then we injected the mixture into mice via the tail vein in 5 seconds. Each mouse received injections once a week for a total of 8 weeks. Mouse tubular fractions were obtained from the kidney cortex using established methods adapted from Yang L *et al*.^36^.

### Statistical analyses

All of the data were expressed as the means ± SD or as medians (Q1–Q3). The data from multiple groups were analyzed with a one-way ANOVA followed by a Tukey’s post hoc test. Data from two groups were compared by t tests. P values < 0.05 were considered significant.

## Supplementary information

Supplementary material
