# Supplementary material for: Upregulated long noncoding RNA LOC105375913 induces tubulointerstitial fibrosis in focal segmental glomerulosclerosis
Source: Sci Rep. 2019 Jan 24;9:716. doi: 10.1038/s41598-018-36902-2 (PMC6345783; doi:10.1038/s41598-018-36902-2)

SUPPLEMENTARY MATERIAL

Title: Upregulated long noncoding RNA LOC105375913 induces tubulointerstitial fibrosis in focal segmental glomerulosclerosis

Author list: Runhong Han[1,2#], Shuai Hu[1#], Weisong Qin[1], Jingsong Shi[1], Caihong Zeng[1], Hao Bao[1*] and Zhihong Liu[1,2*]

Table S1. Oligomers used in this study

Name	Application	Primer list
LOC105375913-F	PCR	CCTGTGCGAAGAAGTTAGCG
LOC105375913-R	PCR	AGAACCTGATCCAGCCCACT
hsa-COL1A1-F	PCR	GAGGGCCAAGACGAAGACATC
hsa-COL1A1-R	PCR	CAGATCACGTCATCGCACAAC
mmu-COL1A1-F	PCR	TAAGGGTCCCCAATGGTGAGA
mmu-COL1A1-R	PCR	GGGTCCCTCGACTCCTACAT
hsa-FN-F	PCR	CGGTGGCTGTCAGTCAAAG
hsa-FN-R	PCR	AAACCTCGGCTTCCTCCATAA
mmu-FN-F	PCR	TTCAAGTGTGATCCCCATGAAG
mmu-FN-R	PCR	CAGGTCTACGGCAGTTGTCA
hsa-ACTB-F	PCR	CTTGACAAAACCTAACTTGCG
hsa-ACTB-R	PCR	TGCTGTCACCTTCACCGTTC
mmu-ACTB-F	PCR	GTGACGTTGACATCCGTAAAGA
mmu-ACTB-R	PCR	GCCGGACTCATCGTACTCC
hsa-pri-miR-27b-F	PCR	CTCCTCCAGAAACCGTGGTC
hsa-pri-miR-27b-R	PCR	GAGGTCATCGCTGGGCATAA
mmu-pri-miR-27b-F	PCR	AACCATGACCTTGGCTGCTCCTG
mmu-pri-miR-27b-R	PCR	GTGACTCCCAATACACTTGACAT
LOC105375913-DNA-sense	pull down	(biotin-) CAATACCACTTGGTGTAAGGAGCCGT
LOC105375913-DNA-antisense	pull down	(biotin-) ACGGCTCCTTACACCAAGTGGTATTG
ChIP-XBP-1s-F	ChIP	TTGCCCTCAGCAAGCAG
ChIP-XBP-1s-R	ChIP	TGATTCAATTACCTCCCACCAG

Table S2. Antibodies used in this study

Antibody	Catalog no.	Company	Reactivity	Application
Snail	3879	CST	Human, Mouse	WB, IHC
Collagen I	14695	Proteintech	Human, Mouse	WB
Fibronectin	15613	Proteintech	Human, Mouse	WB
Phospho-p38	4511	CST	Human	WB
p38	ab31828	Abcam	Human	WB
XBP-1s	83418	CST	Human	WB，CHIP
Phospho-Ser/Thr/Tyr	ADI-905-522	Enzo	Human	WB
β-actin	ab8227	Abcam	Human, Mouse	WB

Supplementary Figure 1: Full length blot for Figure 1

Figure 1g

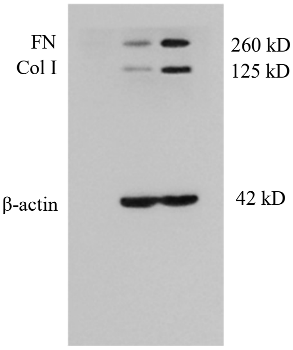

Supplementary Figure 2: Full length blot for Figure 2

Figure 2g

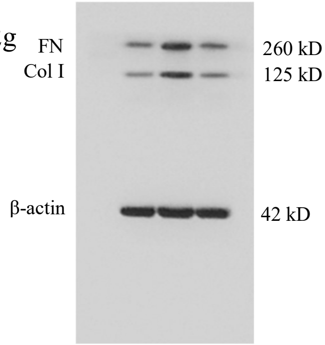

Supplementary Figure 3: Full length blots for Figure 3

Figure 3b

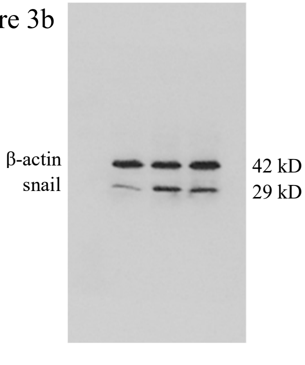

Figure 3d

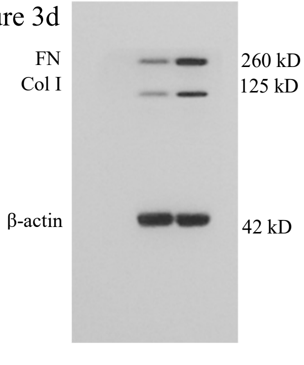

Figure 3f

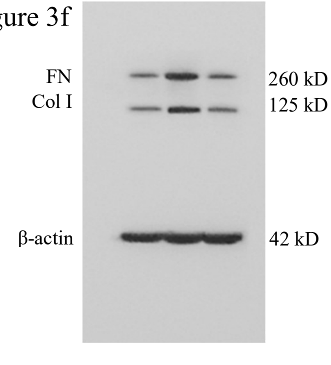

Supplementary Figure 4: Full length blots for Figure 4

Figure 4c

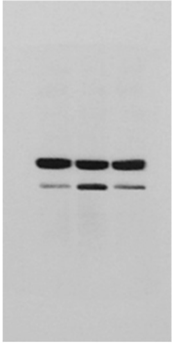

Figure 4d

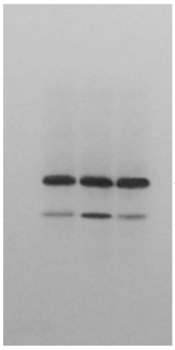

Figure 4e

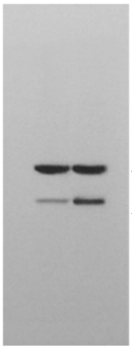

Figure 4k

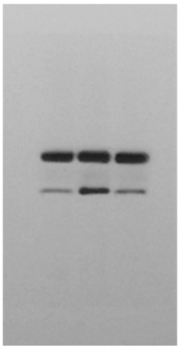

Supplementary Figure 5: Full length blots for Figure 5

Figure 5c

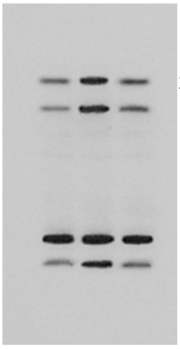

Figure 5d

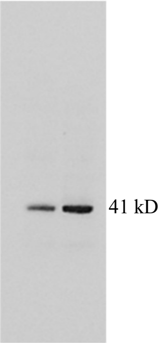

Figure 5d

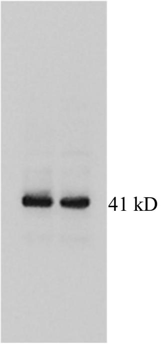

Figure 5e

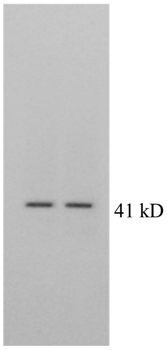

Figure 5e

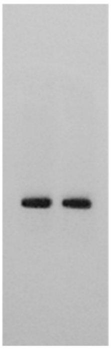

Figure 5h

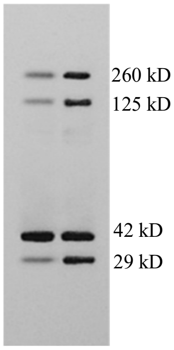

Supplementary Figure 6: Full length blots for Figure 6

Figure 6b

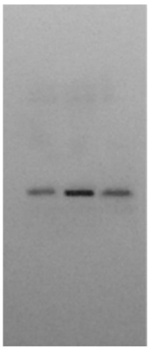

Figure 6b

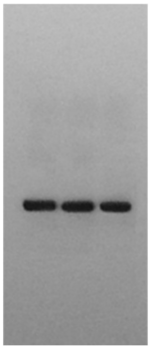

Figure 6h

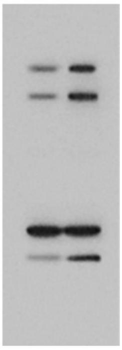

Figure 6j

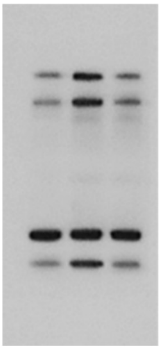

Supplementary Figure 7: Full length blots for Figure 7

Figure 7g

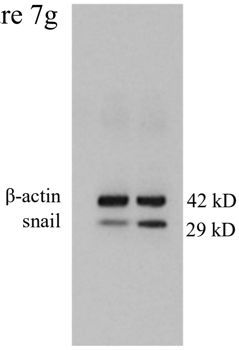

Figure 7i

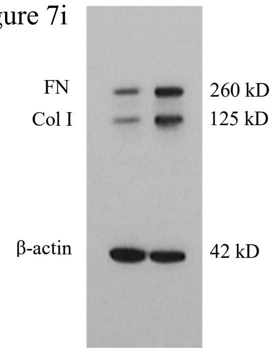

Supplement: Supplementary file 1 — Supplementary material [file 41598_2018_36902_MOESM1_ESM.pdf]
